# *Pseudomonas aeruginosa* PAO1 exopolysaccharides are important for mixed species biofilm community development and stress tolerance

**DOI:** 10.3389/fmicb.2015.00851

**Published:** 2015-08-20

**Authors:** Saravanan Periasamy, Harikrishnan A. S. Nair, Kai W. K. Lee, Jolene Ong, Jie Q. J. Goh, Staffan Kjelleberg, Scott A. Rice

**Affiliations:** ^1^Singapore Centre on Environmental Life Sciences Engineering, Nanyang Technological University, SingaporeSingapore; ^2^Interdisciplinary Graduate School, Nanyang Technological University, SingaporeSingapore; ^3^School of Biological Sciences, Nanyang Technological University, SingaporeSingapore; ^4^School of Biotechnology and Biomolecular Sciences, Centre for Marine Bio-Innovation, University of New South Wales, Sydney, NSWAustralia; ^5^School of Biological, Earth and Environmental Sciences, Centre for Marine Bio-Innovation, University of New South Wales, Sydney, NSWAustralia

**Keywords:** exopolysaccharides, biofilms, mixed species consortia, interspecies competition, stress tolerance

## Abstract

*Pseudomonas aeruginosa* PAO1 produces three polysaccharides, alginate, Psl, and Pel that play distinct roles in attachment and biofilm formation for monospecies biofilms. Considerably less is known about their role in the development of mixed species biofilm communities. This study has investigated the roles of alginate, Psl, and Pel during biofilm formation of *P. aeruginosa* in a defined and experimentally informative mixed species biofilm community, consisting of *P. aeruginosa*, *Pseudomonas protegens*, and *Klebsiella pneumoniae*. Loss of the Psl polysaccharide had the biggest impact on the integration of *P. aeruginosa* in the mixed species biofilms, where the percent composition of the *psl* mutant was significantly lower (0.06%) than its wild-type (WT) parent (2.44%). In contrast, loss of the Pel polysaccharide had no impact on mixed species biofilm development. Loss of alginate or its overproduction resulted in *P. aeruginosa* representing 8.4 and 18.11%, respectively, of the mixed species biofilm. Dual species biofilms of *P. aeruginosa* and *K. pneumoniae* were not affected by loss of alginate, Pel, or Psl, while the mucoid *P. aeruginosa* strain achieved a greater biomass than its parent strain. When *P. aeruginosa* was grown with *P. protegens*, loss of the Pel or alginate polysaccharides resulted in biofilms that were not significantly different from biofilms formed by the WT PAO1. In contrast, overproduction of alginate resulted in biofilms that were comprised of 35–40% of *P. aeruginosa*, which was significantly higher than the WT (5–20%). Loss of the Psl polysaccharide significantly reduced the percentage composition of *P. aeruginosa* in dual species biofilms with *P. protegens* (<1%). Loss of the Psl polysaccharide significantly disrupted the communal stress resistance of the three species biofilms. Thus, the polysaccharide composition of an individual species significantly impacts mixed species biofilm development and the emergent properties of such communities.

## Introduction

Bacteria predominantly occur as biofilms in the environment and biofilm formation is linked to increased tolerance of bacteria to a range of environmental and host related stressors. As a consequence, considerable experimental effort to understand how bacteria regulate biofilm formation and what effectors are involved in the increased resilience of biofilms has been made. Resistance of biofilm cells has been linked in part to the physiological status of the cells, where gradients of nutrients result in a stratified population of cells. Under these conditions the cells within microcolonies are less active or express stationary phase like responses (Hentzer et al., 2005; Waite et al., 2005). Biofilm formation also occurs in response to regulatory processes including quorum sensing or to exposure to stressors such as sub-lethal doses of antibiotics and detergents (Whiteley et al., 2001; Folsom et al., 2010).

One of the defining features of the biofilm is the presence of a self-produced extra-cellular matrix. This matrix not only provides the scaffold for adhesion to surfaces and cohesion between cells, but also protects the cells from stresses such as desiccation, oxidizing agents and host immune defenses (DeVault et al., 1990; Ophir and Gutnick, 1994; Pier et al., 2001; O’Toole, 2003; Parsek and Singh, 2003; Friedman and Kolter, 2004; Jackson et al., 2004; Ryder et al., 2007). The matrix can additionally sequester valuable enzymes and nutrients, cell-to-cell communication signals and fosters the exchange of genetic material (Stoodley et al., 2002). The matrix is typically comprised of a combination of proteins, extracellular DNA and polysaccharides.

The biofilm matrix of *P*. *areuginosa* PAO1 has been shown to include at least three polysaccharides, alginate, Psl, and Pel polysaccharides, and their roles during biofilm development have been demonstrated in biofilm populations (single species systems; Colvin et al., 2011, 2012; Ghafoor et al., 2011; Billings et al., 2013; Zhao et al., 2013). Alginate deficient mutants develop biofilms with a decreased proportion of viable cells and contain significantly more extracellular DNA (Ghafoor et al., 2011). It has also been shown that exposure to oxidative stress induces the overproduction of alginate, which protects the biofilms from oxidative radicals (Mathee et al., 1999; Hentzer et al., 2001). Biofilms of *psl* or alginate deletion mutants failed to form the characteristic mushroom like structures, suggesting these polysaccharides are important for structural development (Ghafoor et al., 2011). Pel was described as being essential for the formation of biofilms by *Pseudomonas aeruginosa* at the air–liquid interface in static broth cultures (Friedman and Kolter, 2004). Psl also plays an important role in the initiation of biofilm formation (Friedman and Kolter, 2004; Jackson et al., 2004; Matsukawa and Greenberg, 2004; Campisano et al., 2006; Ma et al., 2006). More recently, the visco-elastic properties of Pel and Psl were described (Chew et al., 2014), where it was shown that Psl demonstrated properties consistent with elastic materials, suggesting that it is stiff or rigid. In contrast, the Pel polysaccharide was more viscous and was responsible for the formation of biofilm streamers. These properties have been shown to have important outcomes for biofilms that form in industrial settings. For example, biofilms that lack the Psl polysaccharide showed a reduced tendency to inhibit reverse osmosis membrane performance, suggesting that the strong, cohesive properties of Psl were necessary to make an impermeable biofilm (Barnes et al., 2014). Collectively, these data demonstrate that the individual polysaccharide components of the EPS play important roles in biofilm formation and structure development.

While the roles of the matrix components have been well studied in the context of monospecies biofilm development, considerably less is understood about the roles of the matrix in the development of mixed species biofilm communities. This is particularly relevant because in nature, most biofilms are represented by diverse communities rather than populations of single species. For these mixed species biofilm communities, the organization of the different species may be important for community function and therefore, the matrix potentially plays a vital role in the structural organization of mixed species communities. Experiments investigating dual species biofilms formed by *P. aeruginosa* and *Staphylococcus aureus* indicated that the production of Pel and Psl were important for the two bacteria to form biofilms together, suggesting that polysaccharide production may be a key factor in community assembly (Billings et al., 2013; Chew et al., 2014). We have recently established a mixed species biofilm community that results in increased overall biomass of the community relative to single species biofilms formed separately by its members (Lee et al., 2014). Further, the mixed species biofilm demonstrated community level stress protection, which was extended to all of the community members, despite some of those members being individually sensitive to those stresses. The mechanisms that drive community assembly and resistance are currently unknown.

In the present study, we have investigated the role of polysaccharides produced by PAO1 in the establishment of a biofilm community, consisting of *P. aeruginosa* PAO1, *P*. *protegens* Pf-5, and *Klebsiella pneumoniae* KP-1. Specifically, mutants of *P. aeruginosa* that were deficient in the production of alginate, Pel, and Psl or that over expressed alginate, were compared for the formation of mixed species communities. The results demonstrate that the composition of the mixed species biofilm community was strongly influenced by the ability of *P. aeruginosa* to produce the Psl polysaccharide. This highlights the importance of specific polysaccharides in biofilm community assembly and function.

## Materials and Methods

### Bacterial Strains and Culture Media

Bacteria (**Table 1**) were routinely cultured in either M9 minimal medium (48 mM Na_2_HPO_4_; 22 mM KH_2_PO_4_; 9 mM NaCl; 19 mM NH_4_Cl; 2 mM MgSO_4_; 0.1 mM CaCl_2_; and 2 mM glucose) supplemented with 0.2% w/v CAA (supplemented M9 minimal medium), Luria Bertani broth (LB_10_; 10 g L^-1^ NaCl; 10 g L^-1^ tryptone; 5 g L^-1^ yeast extract) or Super Optimal Broth (SOB; 10 mM NaCl; 2.5 mM KCl; 10 mM MgCl_2_; 10 mM MgSO_4_; 20 g L^-1^ tryptone; 5 g L^-1^ yeast extract).

**Table 1:** List of bacterial strains used.

Species and strain	Genotypic and phenotypic characteristics^4^	Source
***Pseudomonas aeruginosa*** PAO1		Lee et al. (2014)
PAO1Δ*alg*	Isogenic *alg8* deletion mutant	Ghafoor et al. (2011)
PAO1Δ*pel*	Isogenic *pelF* deletion mutant	Ghafoor et al. (2011)
PAO1Δ*psl*	Isogenic *pslA* deletion mutant	Ghafoor et al. (2011)
PDO300Δ*mucA*	Mutation in the *mucA22* allele	Mathee et al. (1999)
PAO1-eYFP	Carries the gene encoding eYFP in the intergenic region between coding region of *glmS* and its downstream gene; Gm^R^	Lee et al. (2014)
PAO1Δ*alg*-eYFP		This project
PAO1Δ*pel*-eYFP		This project
PAO1Δ*psl*-eYFP		This project
PDO300Δ*mucA*-eYFP		This project
**^1^*P. protegens* Pf-5**		Lee et al. (2014)
Pf-5-eCFP	Carries the gene encoding eCFP in the intergenic region between coding region of *glmS* and its downstream gene; Gm^R^	Lee et al. (2014)
**^2^*Klebsiella pneumoniae* KP-1**		Lee et al. (2014)
KP-1 -DsRed	Carries the gene encoding DsRedExpress in the intergenic region between coding region of *glmS* and its downstream gene; Gm^R^	Lee et al. (2014)
***Escherichia coli***		
JM109	*end*A1 *gln*V44 *thi*-1 *rel*A1 *gyr*A96 *rec*A1 *mcr*B^+^ Δ(*lac-proAB*) *e14^-^* [F′ *tra*D36 *proAB*^+^ *lac*I^q^ *lacZ*ΔM15] *hsd*R17(r_K_*^-^*m_K_^+^)	Yanisch-Perron et al. (1985)
HPS1	F*^-^* Δ(*lab-proAB*) *end*A1 *gyr*A96 *hsd*R17 *sup*E44 *rel*A1 *rec*A1 *thi rif*^R^ zzx::mini-Tn5Lac4	Choi et al. (2005)
CC118 aaaaaapir	Δ(*ara-leu*) *ara*D Δ*lac*X74 *gal*E *gal*K *pho*A20 *thi*-1 *rps*E *rpo*B *arg*E(*Am*) *rec*Al aaaaaa *pir*	Choi et al. (2005)
DH5α aaaaaapir	F*^-^*,Φ80d*lac*ZΔM15 Δ(*lacZYA-arg*F)U169 *deo*R *rec*A1 *end*A1 *hsd*R17(r_K_*^-^*, m_K_^+^) *pho*A *sup*E44 *thi*-1 aaaaaa *pir*	Choi et al. (2005)
S17-1 aaaaaapir	*hsd*R *rec*A *pro* RP4-2 (Tc::Mu; Km::Tn7; aaaaaa *pir*)	Miller and Mekalanos (1988)
HB101	F*^-^*, *hsd*S20 (rb*^-^*, mb*^-^*), *sup*E44, *ara*14, *gal*K2, *lac*Y1, *pro*A2, *rps*L20 (Str^R^), *xyl*-5, *mtl*-1, l-, *rec*A13, *mcr*A*^-^*, *mcr*B*^-^*	Lambertsen et al. (2004)

*^1^*Pseudomonas fluorescens* Pf-5 has recently been renamed as *P. protegens* Pf-5 (Ramette et al., 2011; Lim et al., 2013)*

*^2^*Klebsiella pneumoniae* is an environmental isolate, has been sequenced and the sequence deposited under the accession number- AVNZ01000000, AVNZ00000000 (Lee et al., 2013).*

*Gm^R^, Gentamicin resistance; Str^R^, Streptomycin resistance.*

### Transformation of *P. aeruginosa* EPS Mutants by Electroporation

Electrocompetent *P. aeruginosa* EPS mutants Δ*mucA*, Δ*alg*, Δ*pel*, and Δ*psl* were prepared as described (Choi et al., 2006). During transformation, the ColE1 replicon-based delivery plasmid and the helper plasmid, pTNS1 (**Table 2**), were added to the electrocompetent cells and electroporated (25 μF, 200 Ω and 2.5 kV cm^-1^) using a Gene Pulser^TM^ apparatus (BIO-RAD, USA). Transformed cells were recovered by the addition of ice cold Super Optimal Broth with Catabolite repression (SOC; SOB supplemented with 2% w/v glucose) and incubated with shaking for 3 h at 37°C. Recovered cells were plated onto LB_5_ agar (5 g L^-1^ NaCl; 10 g L^-1^ tryptone; 5 g L^-1^ yeast extract; 1.5% w/v agar) plates were supplemented with 100 μg mL^-1^ gentamicin for the selection of transformants.

**Table 2:** List of plasmids used in this study.

Plasmid	Relevant characteristic^3^	Source
pTNS1	Helper plasmid, providing the Tn7 transposition function. Ap^R^, R6K *ori*, *ori* T	Choi et al. (2005)
pTNS2	Helper plasmid, providing the Tn7 transposition function. Ap^R^, R6K *ori*, *ori* T	AY884833^1,2^
pTNS2-ColE1	Helper plasmid, providing the Tn7 transposition function. Ap^R^, ColE1 *ori*, *ori* T	Lee et al. (2014)
pUC18T- mini-Tn7T-Gm-eYFP/HPS1	pUC18 –based delivery plasmid for mini-Tn7-Gm-eYFP. Ap^R^, Gm^R^, ColE1 *ori*, *ori*T	DQ493879^1,2^
pUC18TR6K- mini-Tn7T-Gm-eYFP	pUC18 –based delivery plasmid for mini-Tn7-Gm-eYFP. Ap^R^, Gm^R^, R6K *ori*, *ori*T	Lee et al. (2014)
pRK600	Mobilizing plasmid, providing the mobilization ability during conjugation. Ap^R^, Cm^R^, R6K *ori*	Laboratory stock
pUC18TR6K-mini-Tn7T	pUC18 –based vector plasmid for construction of R6K replicon-based delivery plasmids in this project. Ap^R^, R6K *ori*, *ori*T	AY712953^2^

*^1^Plasmids were generously provided by Herbert P. Schweizer (Choi et al., 2005).*

*^2^National Center for Biotechnology Information (NCBI) accession number.*

*^3^Ap^R^, Ampicillin resistance; Cm^R^, Chloramphenicol resistance; Gm^R^, Gentamicin resistance.*

### Determination of Tn7 Insertion Site

Colony PCR was used to verify chromosomal Tn7 insertion using primers specific for the insertion site (**Table 3**) using a C1000^TM^ thermal cycler (BIO-RAD, USA) with an initial denaturation at 97°C for 3 min followed by 35 cycles of amplification (denaturation at 97°C, 30 s; annealing at 55°C, 30 s; extension at 72°C, 1 min) and a final extension at 72°C for 10 min. The PCR product was visualized on a 1% w/v agarose gel and sequenced.

**Table 3:** List of primers used.

Primer	Sequence	Description
ColE1_F	5′AGGATCCCCGGGGATAACGCAGGAAAGAACAT3′	Primer is used during PCR amplification of ColE1 *ori*. Primer is flanked with *Sma*I site at 5′ end.
ColE1_R	5′GATTACGAATTCCTGTCAGACCAAGTTTACTC3′	Primer is used during PCR amplification of ColE1 *ori*. Primer is flanked with *Eco*RI site at 5′ end.
Tn7R	5′CAGCATAACTGGACTGATTTCAG3′	Common primer used for checking chromosomal insertion of Tn7.
PAglmS-down	5′GCACATCGGCGACGTGCTCTC3′	Primer used with Tn7R to check chromosomal insertion of Tn7 in PAO1

### Flow Cells Dynamics Experiments

Biofilms were cultivated in three-channel flow cells (channel dimensions, 1 mm × 4 mm × 40 mm; Biocentrum-DTU, 2005; Sternberg and Tolker-Nielsen, 2005). The flow cells were supplied with supplemented M9 minimal medium at 9 mL h^-1^ (mean velocity = 0.625 mm s^-1^) with a Reynolds number of 1.12. Each channel was injected with 0.5 mL of diluted overnight culture containing approximately 1 × 10^8^ cfu mL^-1^. Mixed species biofilms were established by inoculating mixed cultures of PAO1 EPS mutants, Pf-5, and KP-1 in the ratio of 5:5:1, respectively.

### SDS Treatment

Flow cells biofilms were grown in M9 supplemented with 2 mM glucose and 0.2% w/v CAA. After 3 days, biofilms were treated with M9 glucose, CAA, and 0.1% SDS under flow conditions for 2 h. Images were collected before and after the treatment for the quantification of biomass.

### Microscopy, Image and Statistical Analysis

All microscopic observations and image acquisition were performed using a CLSM (LSM 780, Carl Zeiss, Germany). For each channel, five image stacks were acquired, covering a total area of approximately 9 × 10^5^ μm^2^, which was more than the suggested minimum of 1 × 10^5^ μm^2^ to acquire representative data (Korber et al., 1993). For image analysis, a total of 15 image stacks (five from each experiment) were quantified for each biofilm type using IMARIS (Bitplane AG, Switzerland). Statistical analysis was performed using Graph pad PRISM.

## Results

### The Role of Pel, Psl, and Alginate in the Development of Three-Species Biofilm Communities

**FIGURE 1:**
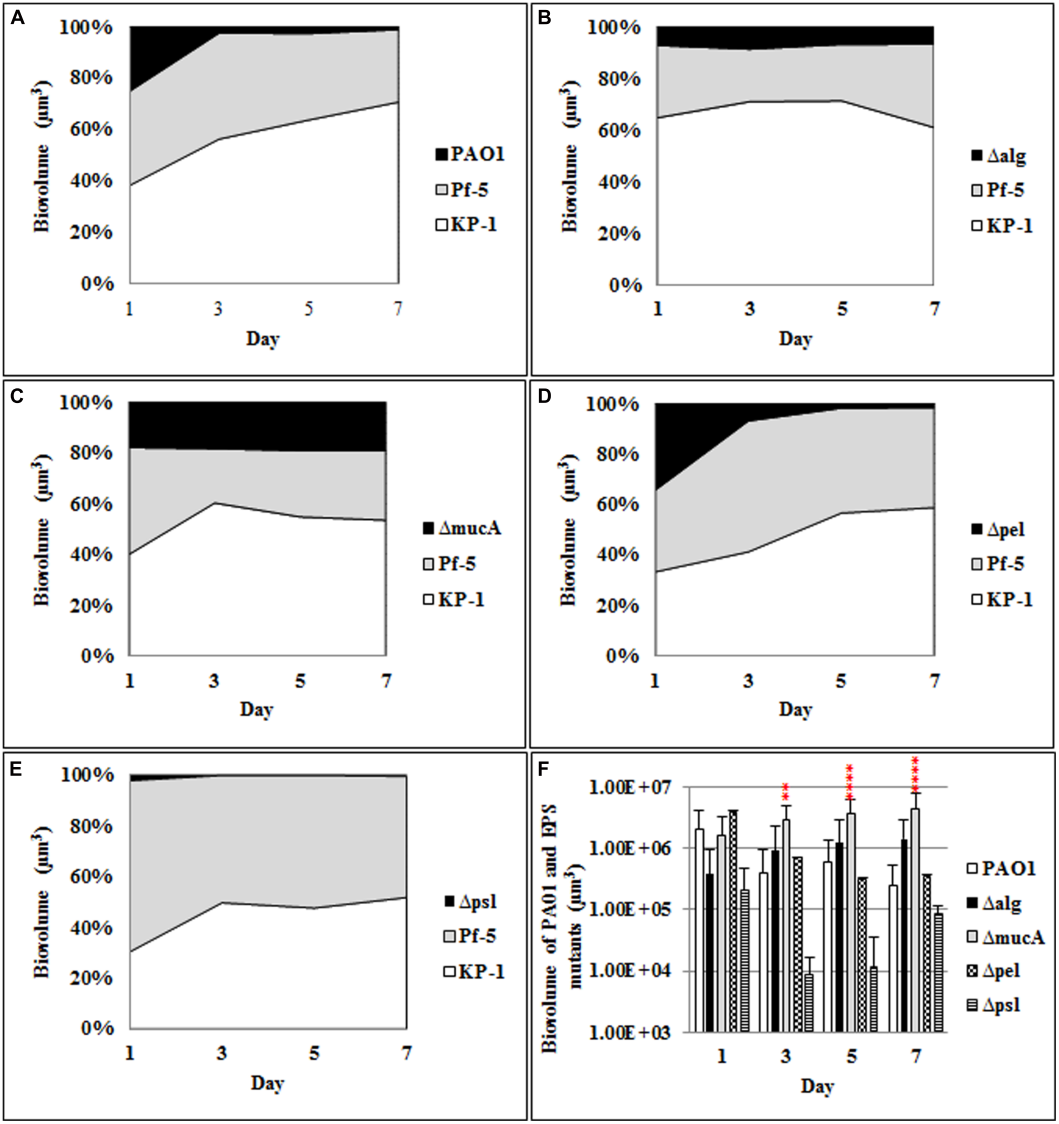
**Spatial and temporal development of *Pseudomonas aeruginosa* polysaccharide mutants grown with *P. protegens* and *Klebsiella pneumoniae* as three species biofilms.** The proportion of the three species within the mixed species over the 7 days period was determined by quantitative image analysis. **(A)** *P. aeruginosa* wild-type (WT), **(B)** Δ*alg*, **(C)** Δ*mucA*, **(D)** Δ*pel*, **(E)** Δ*psl*, and **(F)** biovolumes of *P. aeruginosa* WT and polysaccharide mutants. Statistical analysis was performed vs. the corresponding WT samples grown in parallel **P < 0.01, ****P < 0.0001.

To determine the roles of alginate, Psl, and Pel produced by *P. aeruginosa* in mixed species biofilm community development, polysaccharide mutants of *P. aeruginosa*, *alg*, *mucA*, *pel*, and *psl* were cultivated with *P. protegens* and *K. pneumoniae* as triple species biofilms. Initial attachment of the alginate overproducing strain, *mucA*, was similar to the wild-type (WT) *P. aeruginosa* (**Figure 1**; **Supplementary Figure S1**). However, in contrast to the WT *P. aeruginosa*, the biovolume of the *mucA* mutant remained constant at 20% throughout the duration of the experiment, which was significantly higher than the WT (2%). Mutants in the *alg* and *pel* polysaccharide genes showed an increase in the amount of *P. aeruginosa* present in the three species biofilm community during the initiation of biofilm formation (**Figures 1B,D,F**). When the *alg* mutant was included in the biofilm, the architecture of *P. protegens* changed from one dominated by microcolonies to a more filamentous biofilm and the *alg* mutant completely covered the top of the biofilm at day 7 (**Supplementary Figure S1**). In contrast, the *psl* mutant was below the detection level in the triple species biofilms (**Figures 1E,F**) with *P. protegens* and *K. pneumoniae* accounting for 52.24 and 47.69% of the biofilm biomass, respectively (**Figure 1E**).

### The Role of Pel, Psl and Alginate in Dual Species Biofilm Development

**FIGURE 2:**
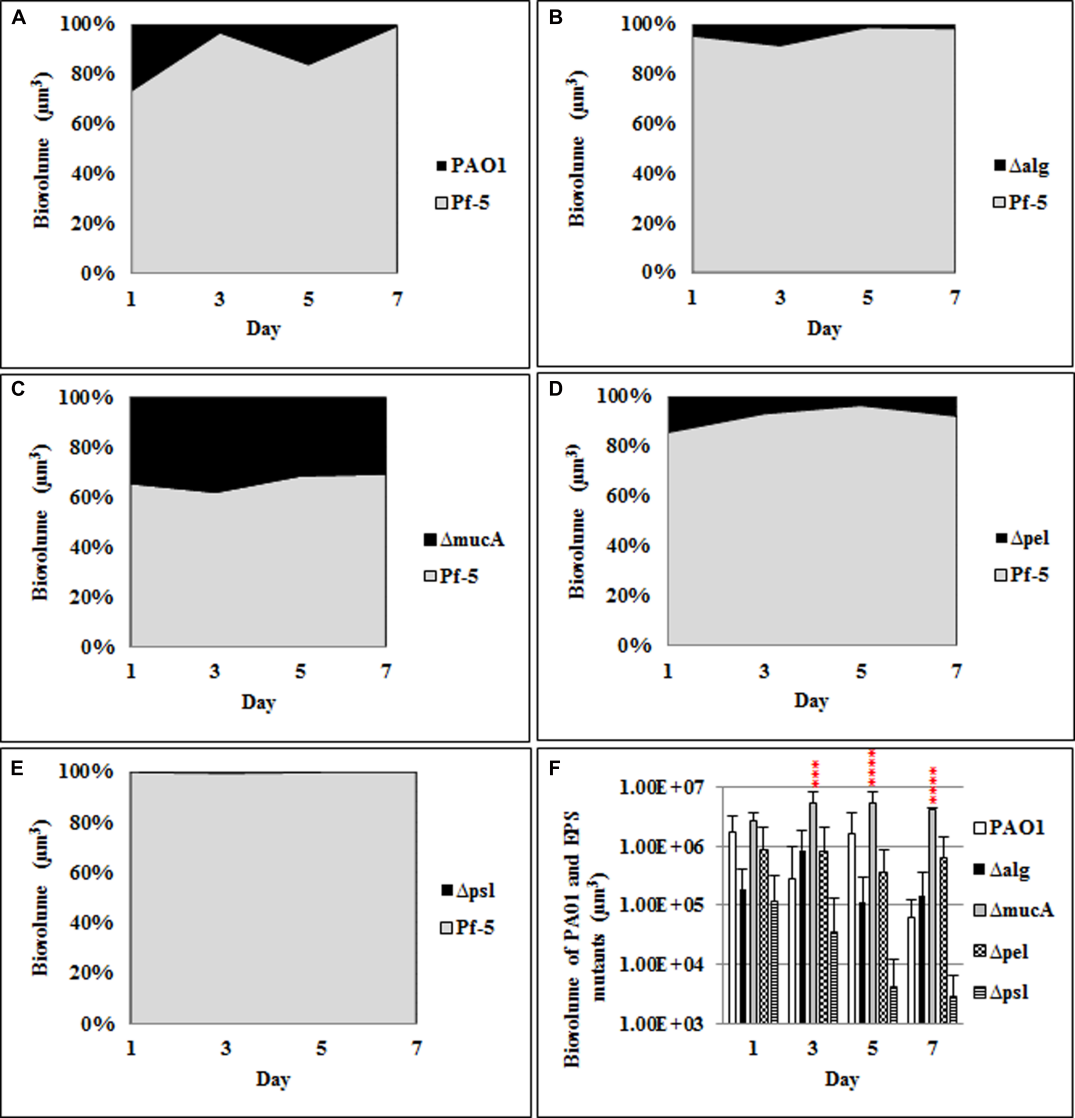
**Spatial and temporal development of *P. aeruginosa* polysaccharide mutants grown with *P. protegens* as dual species biofilms**. The proportion of the two species was calculated by quantitative image analysis. **(A)** *P. aeruginosa* WT, **(B)** Δ*alg*, **(C)** Δ*mucA*, **(D)** Δ*pel*, **(E)** Δ*psl*, and **(F)** biovolumes of *P. aeruginosa* and polysaccharide mutants. Statistical analysis was performed vs. the corresponding WT samples grown in parallel, which were very similar in all cases ***P < 0.001, ****P < 0.0001.

Similarly, the roles of the *P. aeruginosa* polysaccharides in mediating dual species biofilm interactions were also investigated. When grown as a dual species biofilm with *P. protegens* (**Figure 2**; **Supplementary Figure S2**) the *mucA* mutant showed a significant increase (35–40%) in relative biovolume compared to the WT *P. aeruginosa* (5–20%). There was no significant difference in the biovolumes for the *pel* and *alg* mutants relative to the WT. As observed for the three species biofilm, the *psl* mutant (<1% biovolume) was also severely impaired in its ability to establish a dual species biofilm with *P. protegens*.

**FIGURE 3:**
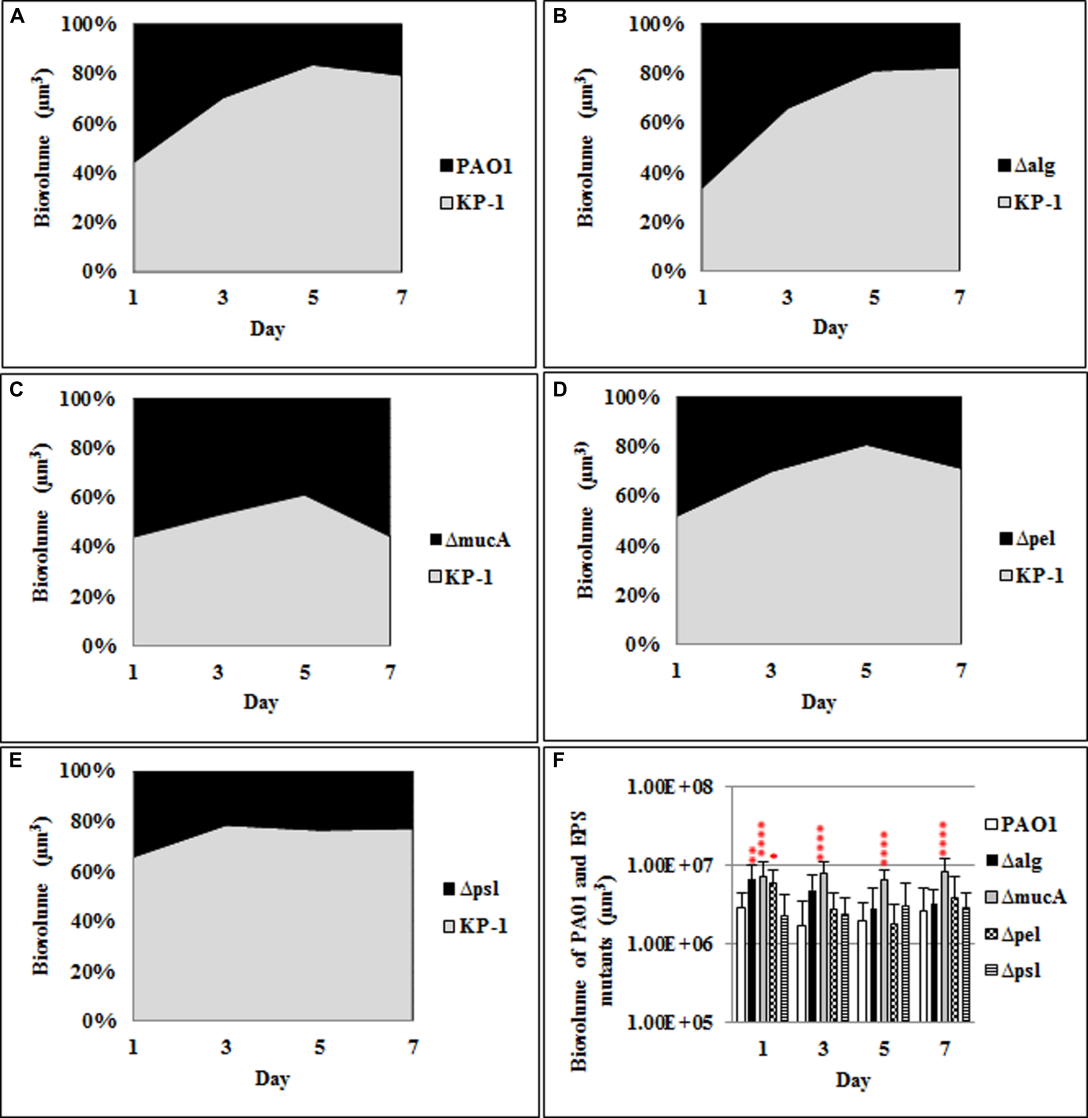
**Spatial and temporal development of *P. aeruginosa* polysaccharide mutants grown with *K. pneumoniae* as dual species biofilms**. The proportion of the two species was calculated by quantitative image analysis. **(A)** *P. aeruginosa* WT, **(B)** Δ*alg*, **(C)** Δ*mucA*, **(D)** Δ*pel*, **(E)** Δ*psl*, and **(F)** biovolumes of *P. aeruginosa* and polysaccharide mutants. Statistical analysis was performed vs. the corresponding WT samples grown in parallel, which were very similar in all cases *P < 0.01, **P < 0.001, ****P < 0.0001.

When the polysaccharide mutants formed dual species biofilms with *K. pneumoniae* (**Figure 3**), *alg* and *pel* mutants exhibited significant increases at day 1, but not for the remainder of the experiment, relative to the WT *P. aerugionsa*. There was a statistically significant increase in the biofilm biomass of the alginate over producing strain, *mucA*, for days 3–7 of biofilm development (39–47%) relative to the WT *P. aeruginosa* (16–30%).

The data suggest that the mucoid strain of *P. aeruginosa* is better able to compete in mixed species biofilm communities while the *psl* mutant is generally less fit under these conditions. The primary changes in *P. aeruginosa* biofilm biomass were observed when it was grown with *P. protegens* suggesting the resource competition in the mixed species biofilms is strongest between these two closely related species.

### The Role of Polysaccharides in the Stress Resistance of Mixed Species Biofilms

**FIGURE 4:**
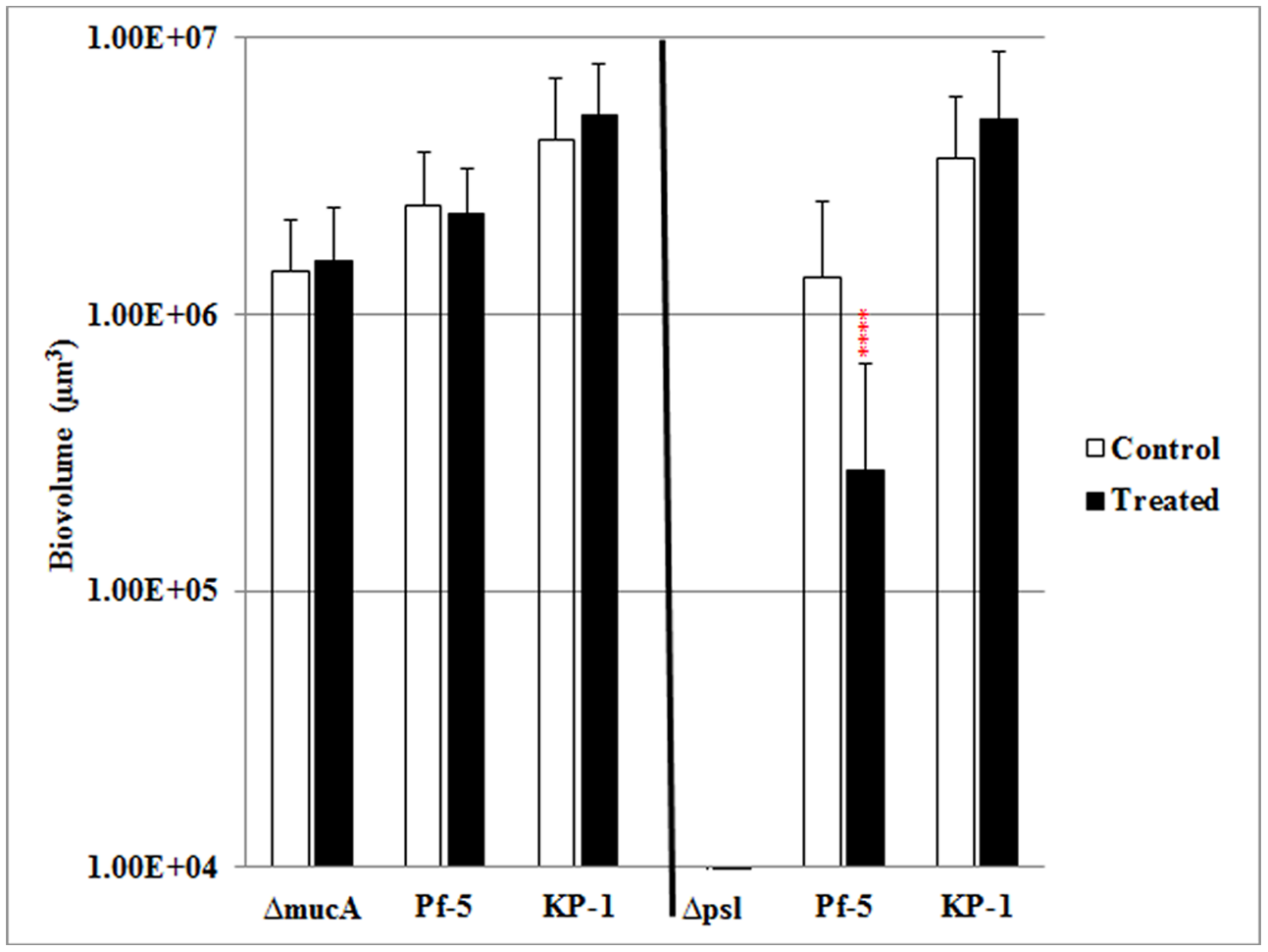
**The role of alginate and Psl in stress resistance of mixed species biofilms.** Three species biofilms were formed for 4 days and exposed to 0.1% SDS for 2 h. The biofilm biovolumes of *P. aeruginosa mucA* **(left)**, *psl* **(right)**, Pf-5, and KP-1 were determined by quantitative image analysis before and after SDS treatment. Statistical analysis was performed vs. the corresponding WT samples grown in parallel, which were very similar in all cases ****P < 0.0001.

It was previously shown that this mixed species biofilm community displays enhanced resistance to SDS and antibiotic stress relative to biofilms formed by the individual species alone (Lee et al., 2014). Further, the stress resistance was a communal property, where all three species were equally protected, despite monospecies biofilms of *P. protegens* being highly sensitive to SDS exposure. To determine the role of the polysaccharide component of the EPS in stress resistance of mixed species biofilms, mutants that either overproduce alginate, *mucA*, or that were defective for the production of Psl were tested for their contribution to the SDS resistance of the three species biofilms. These two strains were used since the *mucA* strain showed an increased proportion in the mixed species biofilm, while the *psl* mutant was less competitive during mixed species growth. Mixed species biofilms formed with the *mucA* mutant showed similar protection as the WT *P. aeruginosa* and protection was shared across all three species (**Figure 4**). In contrast, mixed species biofilms that included the *psl* mutant showed a significant reduction in biofilm biomass after SDS stress. The biomass of *P. protegens* was reduced by fivefold, indicating that it was no longer protected during mixed species biofilm growth. The biomass of *K. pneumoniae* showed similar amounts of biofilm before and after surfactant exposure and hence was unaffected by the change in biofilm composition.

## Discussion

The dynamics of biofilm formation are influenced by a number of biotic and abiotic factors (Costerton et al., 1994; Wolfaardt et al., 1994). While the effects of polysaccharides on biofilm development have been well studied for biofilm populations, less is understood about their role during the development of mixed species biofilm communities. We have investigated here the contribution of the three known polysaccharides produced by *P. aerugionsa* to determine their role in mixed species biofilm development. It was observed that the initial attachment of the mucoid *P. aerugionsa mucA* mutant was higher than for the WT, as evidenced by the increased proportion of the mutant in the mixed species biofilm at day 1 and for the remainder of the biofilm development cycle. This effect was seen when the *mucA* strain was present in the three species as well as dual species biofilms. Alginate over expression in *P. aerugionsa* is frequently associated with chronic lung infections and mucoid strains have been shown to have increased resistance to stressors. Here we observed that alginate over production resulted in an increase in the biofilm biomass of *P. aeruginosa* relative to *P. protegenes* and *K. pneumoniae*. Therefore, over production of alginate could enhance the competitive fitness of *P. aerugionsa* during mixed species biofilm formation during chronic lung infection.

The *pel* mutant was similar to the WT in its contribution to the biofilm and thus, under these conditions, may play a lesser role in mixed species biofilm development. While Pel was not essential, loss of this polysaccharide resulted in alteration of the biofilm structure. It was observed that the height of microcolonies formed by the *pel* mutant ranged from 40 to 50 μm compared to the WT microcolonies, for which the microcolony heights ranged from 70 to 80 μm (data not shown). It has also been shown that loss of Pel from *P. aeruginosa* biofilms results in stiffer, more rigid biofilms (Chew et al., 2014). Therefore, the loss of Pel may stiffen the mixed species biofilm, preventing the expansion of microcolony formation. In dual species biofilms of *P. aeruginosa* and *S. aureus*, Pel was shown to be essential for the two species to form mixed, integrated biofilm communities (Chew et al., 2014).

In contrast to the *pel* mutant, the *psl* mutant was almost completely excluded from both triple and dual (*P. aeruginosa* + *P. protegens*) mixed species biofilms. This observation is in agreement with the role of Psl in monospecies biofilm formation, where loss of the polysaccharide results in a severe defect in biofilm formation (Ghafoor et al., 2011). During attachment, Psl is anchored on the cell surface in a helical pattern, which promotes cell–cell interactions and assembly of a matrix, to hold the bacteria in the biofilm and on the surface (Ma et al., 2009). When grown with *S. aureus*, the Psl mutant formed well mixed dual species biofilms (Chew et al., 2014), further supporting the role of Psl as a rigid polymer responsible for the formation of stiff, inflexible microcolonies.

Given that mixed species biofilms display enhanced stress resistance (Lee et al., 2014) and that the polysaccharide Psl has been shown to play a role in the protection of other species in biofilm communities, the roles of alginate and Psl in the surfactant stress response of mixed species biofilms were tested. Previously, it was shown that monospecies biofilms of *P. protegens* were sensitive to SDS stress, but when *P. protegens* was grown as a biofilm with *P. aeruginosa* and *K. pneumoniae*, it was protected in the mixed species biofilm. When the WT *P. aeruginosa* was replaced with the *mucA* mutant, the all of the community members were equally protected during mixed species biofilm growth. In contrast, when the mixed species biofilm included the *psl* mutant, the protection was lost and both *P. aeruginosa* and *P. protegens*, showed a significant decrease after exposure to SDS stress. This observation suggests that Psl is required for community level protection against SDS stress. Similarly, it was previously shown that Psl plays a role in mediating antibiotic resistance of *P. aeruginosa* biofilms and that the antibiotic resistance afforded by Psl could also protect *Escherichia coli* and *S. aureus* when grown as co-culture biofilms with *P. aeruginosa* (Billings et al., 2013). Thus, Psl may play a more general role in mediating stress tolerance of mono and mixed species biofilms, hence providing protection of biofilm populations as well as communities.

## Conclusion

The data presented here show that specific polysaccharides, such as Psl and alginate play important roles for *P. aeruginosa* during mixed species biofilm growth. The production of these polysaccharides not only impact the competitive fitness of a species during mixed biofilm growth, but also has significant effects on the function of that community. Therefore, biofilm matrix biomolecules may individually play significant roles in the formation of biofilm communities, arguably the natural state of most biofilm systems, and these functions may not be evident from population based studies.

## Conflict of Interest Statement

The authors declare that the research was conducted in the absence of any commercial or financial relationships that could be construed as a potential conflict of interest.

## Supplementary Material

Figure S1 **Ortho views of confocal micrographs of mixed species biofilms composed of *Pseudomonas aeruginosa* polysaccharide mutants (yellow), *P. protegens* (blue) and *Klebsiella pneumoniae* (red) grown on M9 supplemented with 2 mM glucose + 0.2% CAA. (A)** *P. aeruginosa* wild-type (WT), **(B)** Δ*alg*, **(C)** Δ*mucA*, **(D)** Δpel, and **(E)** Δ*psl*. The top and side images of each panel represent the x–z and y–z planes, respectively. The green and red lines indicate the positions corresponding to the x–z and y–z cross sections, respectively. The blue line indicates the x–y plane of the main panel. Magnification 200×.

Figure S2 **Dual species biofilms comprised of *P. aeruginosa* polysaccharide EPS mutants and *P. protegens* grown on 2 mM glucose + 0.2% CAA**. The ortho view of confocal micrographs of dual species composed of *P. aeruginosa* polysaccharide mutants (yellow) and *P. protegens* (blue) imaged over 7 days. The top and side images of each panel represent the x–z and y–z planes, respectively. The green and red lines indicate the positions corresponding to the x–z and y–z cross sections, respectively. The blue line indicates the x–y plane of the main panel. Magnification 200×.

Figure S3 **Dual species biofilms of *P. aeruginosa* polysaccharide mutants and *K. pneuomoniae* grown in 2 mM glucose + 0.2% CAA**. The ortho view of confocal micrographs of dual species composed of *P. aeruginosa* polysaccharide mutants (yellow) and *K. pneumoniae* (red) imaged over 7 days. The top and side images of each panel represent the x–z and y–z planes, respectively. The green and red lines indicate the positions corresponding to the x–z and y–z cross sections, respectively. The blue line indicates the x–y plane of the main panel. Magnification 200×.
