# Supplementary figures and images for: Pseudomonas aeruginosa PAO1 exopolysaccharides are important for mixed species biofilm community development and stress tolerance
Source: Front Microbiol. 2015 Aug 20;6:851. doi: 10.3389/fmicb.2015.00851 (PMC4542536; doi:10.3389/fmicb.2015.00851)

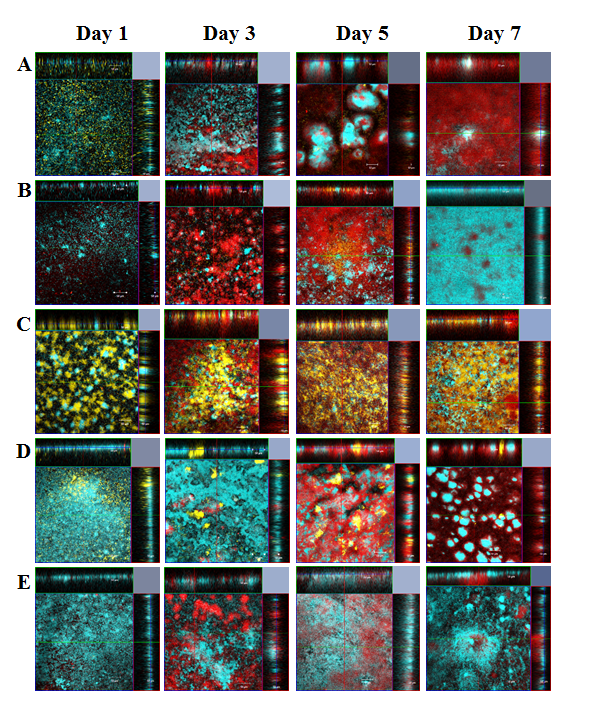

Supplement: Figure S1 — Ortho views of confocal micrographs of mixed species biofilms composed of Pseudomonas aeruginosa polysaccharide mutants (yellow), P. protegens (blue) and Klebsiella pneumoniae (red) grown on M9 supplemented with 2 mM glucose + 0.2% CAA. (A) P. aeruginosa wild-type (WT), (B) Δalg, (C) ΔmucA, (D) Δpel, and (E) Δpsl. The top and side images of each panel represent the x–z and y–z planes, respectively. The green and red lines indicate the positions corresponding to the x–z and y–z cross sections, respectively. The blue line indicates the x–y plane of the main panel. Magnification 200×. [file Image_1.TIF]

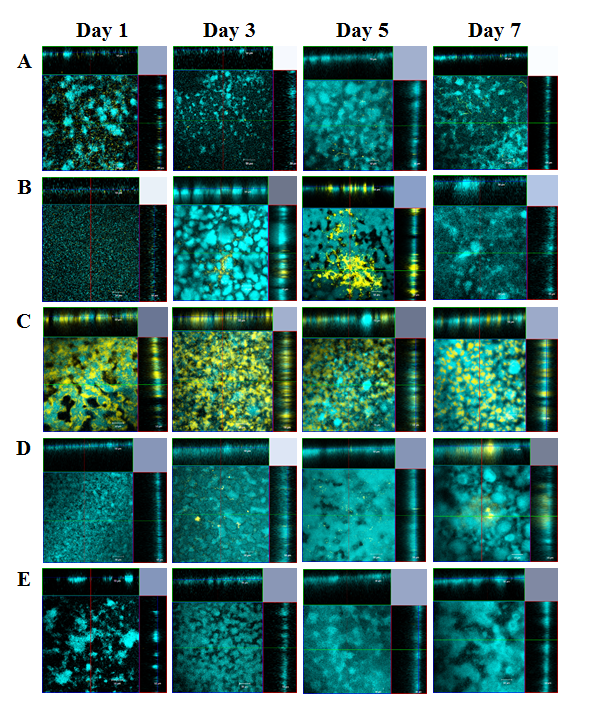

Supplement: Figure S2 — Dual species biofilms comprised of P. aeruginosa polysaccharide EPS mutants and P. protegens grown on 2 mM glucose + 0.2% CAA. The ortho view of confocal micrographs of dual species composed of P. aeruginosa polysaccharide mutants (yellow) and P. protegens (blue) imaged over 7 days. The top and side images of each panel represent the x–z and y–z planes, respectively. The green and red lines indicate the positions corresponding to the x–z and y–z cross sections, respectively. The blue line indicates the x–y plane of the main panel. Magnification 200×. [file Image_2.TIF]

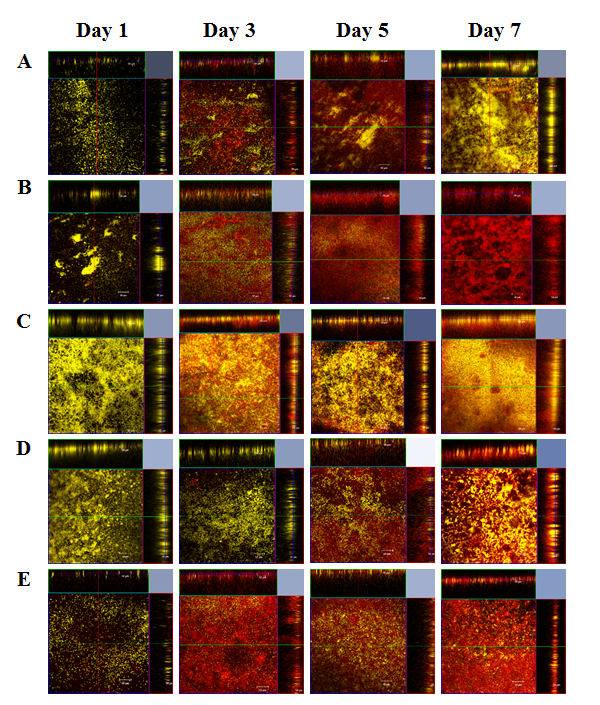

Supplement: Figure S3 — Dual species biofilms of P. aeruginosa polysaccharide mutants and K. pneuomoniae grown in 2 mM glucose + 0.2% CAA. The ortho view of confocal micrographs of dual species composed of P. aeruginosa polysaccharide mutants (yellow) and K. pneumoniae (red) imaged over 7 days. The top and side images of each panel represent the x–z and y–z planes, respectively. The green and red lines indicate the positions corresponding to the x–z and y–z cross sections, respectively. The blue line indicates the x–y plane of the main panel. Magnification 200×. [file Image_3.TIF]
